# *Chrysospleniumramosissimum* Y.I.Kim & Y.D.Kim (Saxifragaceae), a new species from Korea

**DOI:** 10.3897/phytokeys.111.27182

**Published:** 2018-11-06

**Authors:** Yong-In Kim, Seong-Hyun Cho, Jung-Hoon Lee, Dae-Hyun Kang, Jin Hee Park, Young-Dong Kim

**Affiliations:** 1 International Biological Material Research Center, Korea Research Institute of Bioscience and Biotechnology, Daejeon 34141, South Korea; 2 Multidisciplinary Genome Institute, Life Science Hall, Hallym University, Chuncheon, Gangwon 24252, South Korea; 3 Freshwater Bioresources Research Division, Nakdonggang National Institute of Biological Resources, Sangju, Gyeongbuk 37242, South Korea; 4 Department of Life Sciences, Hallym University, Chuncheon, Gangwon 24252, South Korea

**Keywords:** *
Chrysosplenium
*, endemic species, seed morphology, sterile branch, DNA barcode

## Abstract

This study describes and illustrates *Chrysospleniumramosissimum*, a new plant species from Mt. Seonjaryeong, located in the central region of the Korean Peninsula. The species is most similar to *C.valdepilosum* but is readily distinguishable by the presence of yellowish-green bracteal leaves during flowering, highly branched sterile branches, shiny silvery dots on sterile branch leaves and larger tubercles on the seed coat.

## Introduction

*Chrysosplenium* L. is a distinct genus belonging to the family Saxifragaceae, as it possesses tetramerous flowers and petaloid sepals (Bensel and Palser 1975; Soltis 2007; Soltis et al. 2001). This genus is primarily restricted to the northern hemisphere except for two species in Chile, with species occurring in eastern North America (two species), western North America (four species), Europe (two species) and eastern Asia, where the greatest number of species are present, numbering approximately 50 (Hara 1957; Spongberg 1972). Although estimates of the number of taxa are controversial due to its complex taxonomy, approximately 70 taxa from the genus are recognised worldwide (Maximowicz 1859, 1872, 1877, 1879; Hara 1957; Spongberg 1972; Pan 1986; Ye and Zhang 1994; Wakabayashi and Ohba 1995; Wakabayashi and Takahashi 1999; Han et al. 2011; Bhaumik 2014; Kim 2014; Kim and Kim 2015; Liu et al. 2016; Wakabayashi et al. 2018).

*Chrysosplenium* has been classified into two sections and 17 section (Hara 1957), of which nine species representing two sections and five section are distributed in Korea (Chung and Kim 1988; Kim and Kim 2011, 2105; Han et al. 2011, 2012). The section *Pilosa* Maxim. is known to be endemic to northeast Asia and consists of approximately 20 taxa (Franchet and Savatier 1878; Nakai 1914; Kitagawa 1934; Ohwi 1934; Hara 1957; Pan 1986; Wakabayashi and Takahashi 1999; Han et al. 2011; Kim and Kim 2015). The section is characterised by yellow or white erect sepals, opposite leaves and pilose stems (Hara 1957). Currently, five species of the section are recognised in Korea (Kim 2014): *C.aureobracteatum* Y.I. Kim & Y.D. Kim, *C.barbatum* Nakai, *C.epigealum* J.W. Han & S.H. Kang, *C.flaviflorum* Ohwi and *C.valdepilosum* (Ohwi) S.H. Kang & J.W. Han.

During a floristic survey of Mt. Seonjaryeong, located in Pyeongchang-gun, Gangwon-do, Korea in August of 2014, we collected a species of *Chrysosplenium* with a distinct stem feature (i.e. highly branched sterile branches). Additional fieldwork was conducted from April through July 2015 to collect flowering individuals and seeds for specimen and morphological examinations. After consulting relevant literature on *Chrysosplenium* (Franchet and Savatier 1878; Nakai 1914; Kitagawa 1934; Ohwi 1934; Hara 1957; Pan 1986; Han et al. 2011; Kim and Kim 2015) and examining herbarium specimens at HHU, TI, KB, KH, KWNU, KUS, IUI, KYO and PE, as well as images of type specimens available at the Global Plants website at JSTOR (https://plants.jstor.org), we recognised that the taxon belongs to the section *Pilosa*. Upon further examination, the plant was distinguished from all known species of the section based on morphological characters. The species is most similar to *C.valdepilosum*, which has been considered a variety of *C.pilosum* but recently recognised as a distinct species (Kim and Kim 2011, Han et al. 2011). The new species, however, is readily distinguishable by the presence of yellowish-green bracteal leaves during flowering, highly branched sterile branches, shiny silvery dots on sterile branch leaves and larger tubercles on the seed coat. This leads us to the conclusion that it represents an undescribed species. Here, the new species is described and illustrated.

## Materials and method

### Morphological observations

Photographs of the habit and macro-morphological characters were taken in the field. Morphological observations and measurements of the new species, based on living and dry plant specimens and preserved materials, were carried out. All morphological characters were observed and photographed with a Zeiss Stemi SV 11 Apo stereoscopic microscope and a Zeiss AxioCam MRc 5 microscope camera. Seed coat characters were revealed by a Hitachi S-3400N scanning electronic microscope.

## Taxonomic treatment

### Chrysosplenium ramosissimum

Taxon classificationPlantaeSaxifragalesSaxifragaceae

Y.I.Kim & Y.D.Kim sp. nov.

urn:lsid:ipni.org:names:60477346-2

**Figure 1.**
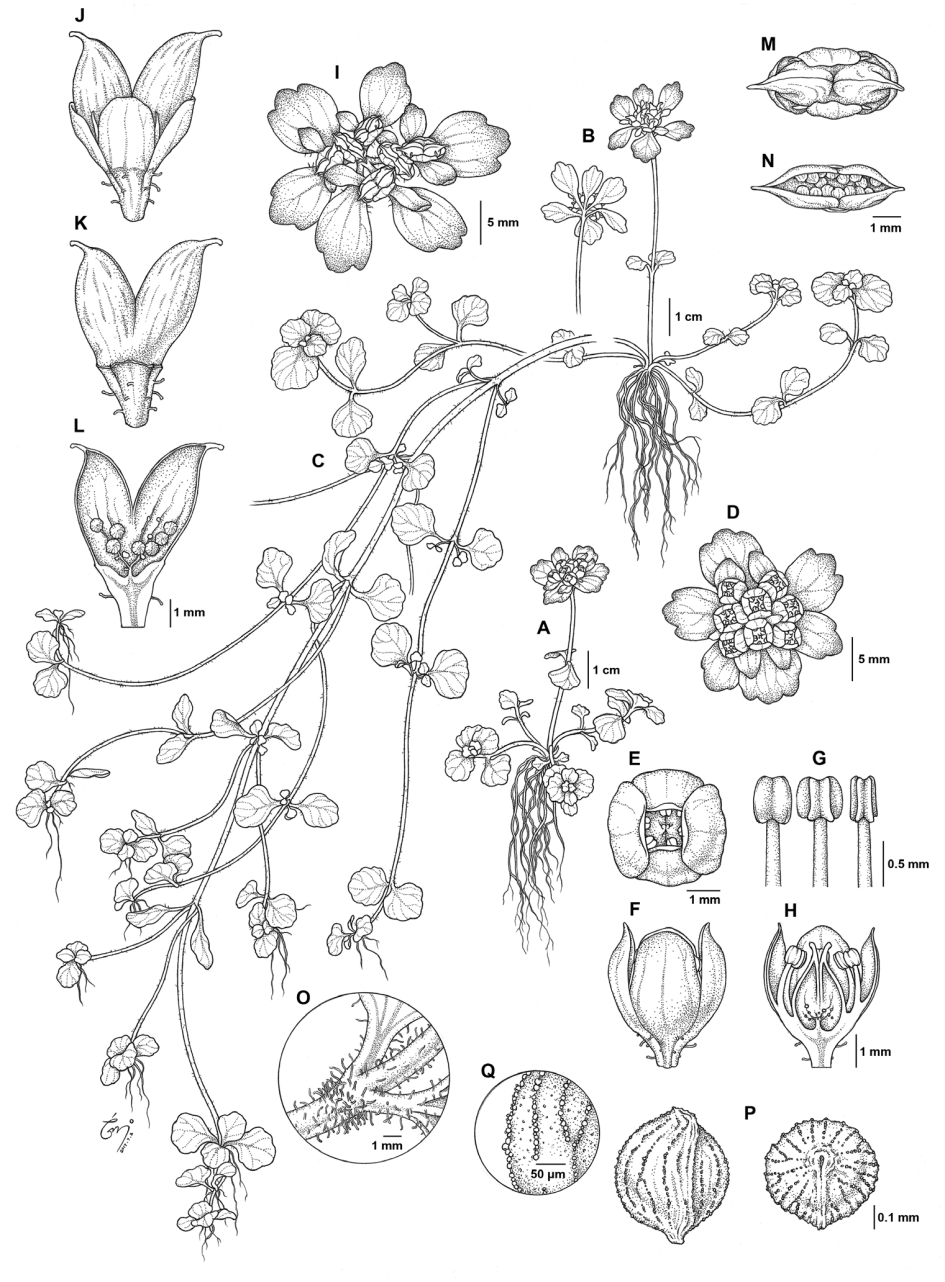
*Chrysospleniumramosissimum* Y.I.Kim & Y.D.Kim. **A** Flowering individual **B** fruiting individual **C** sterile branch habit after fruiting **D** inflorescence and bracteal leaves **E–F** flower **G** stamen at various stages **H** flower longitudinal section **I** infructescence and bracteal leaves **J** capsule with persistent sepals **K** capsule, sepals removed **L** capsule, longitudinal section **M** capsule, before dehiscence (top view) **N** capsule, after dehiscence (top view) **O** node of sterile branch, enlarged **P** seed, side view (left), top view (right) **Q** seed coat, enlarged.

**Figure 2.**
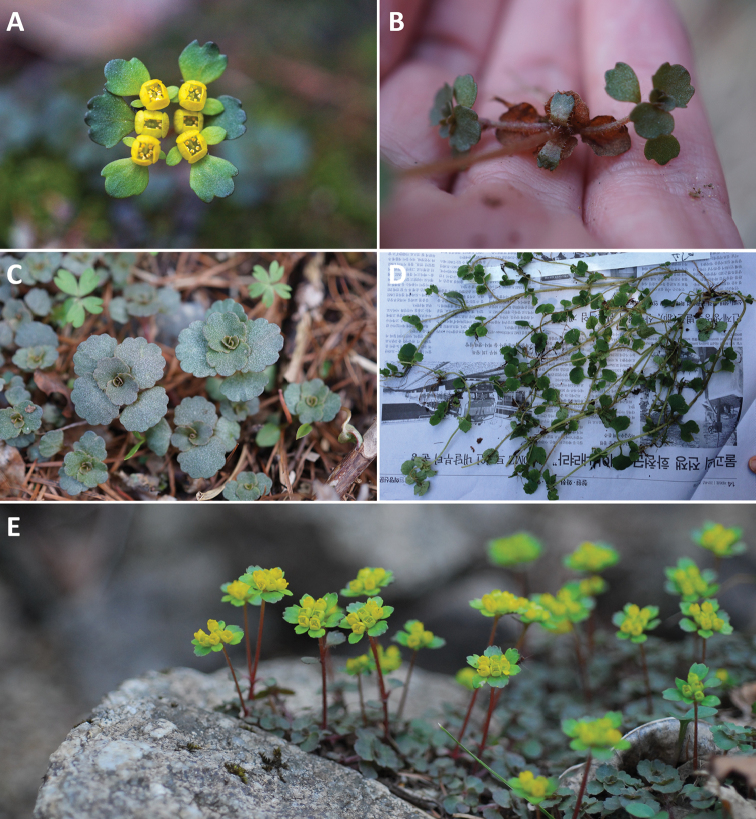
*Chrysospleniumramosissimum* Y.I.Kim & Y.D.Kim. **A** Inflorescence with bracteal leaves **B** sterile branches and basal leaves during flowering with withered basal leaves **C** sterile branch leaves with shiny silvery spots during flowering **D** sterile branch after fruiting **E** plant habit during flowering.

**Figure 3.**
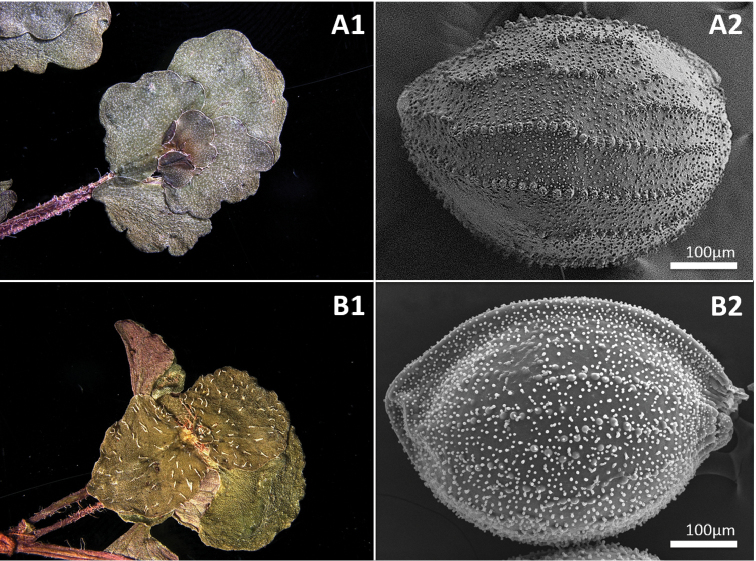
Upper surface of sterile branch leaves of *Chrysospleniumramosissimum* (**A1**) and *C.valdepilosum* (**B1**). Scanning electron micrograph of seeds of *C.ramosissimum* (**A2**) and *C.valdepilosum* (**B2**).

Figs 1 , 2 , 3A1, A2 , 4A 가지털괭이눈(Ga-ji-teol-gwaeng-i-nun)

#### Diagnosis.

**Figure 4.**
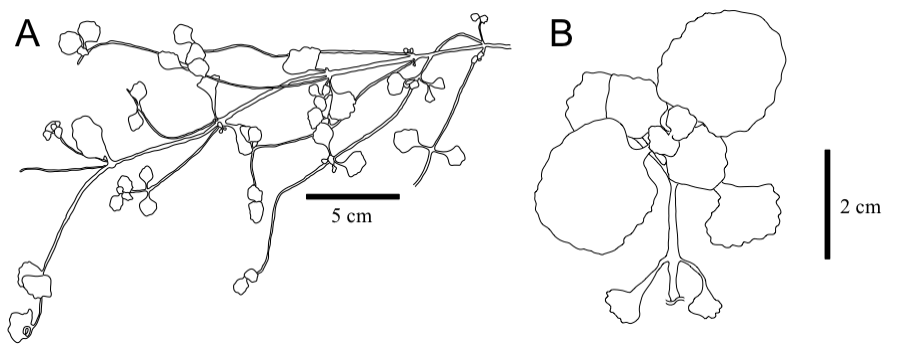
Sterile branch outline of *Chrysospleniumramosissimum* (**A**) and *C.valdepilosum* (**B**) after fruiting.

*Chrysospleniumramosissimum* is most similar to the sympatric species *C.valdepilosum*, but the former is readily distinguishable by the presence of yellowish-green (vs. bright yellow) bracteal leaves during flowering, highly branched and elongated sterile branches after fruiting (Fig. 4), shiny silvery dots on sterile branch leaves and larger tubercles on the seed coat (Fig. 3).

#### Type.

SOUTH KOREA. Gangwon-do: along a stream near a hiking trail to Guksa Seonghwangsa (temple), Mt. Seonjaryeong, Hoenggye-ri, Daegwallyeong-myeon, Pyeongchang-gun, 37°41'25.80"N, 128°45'27.22"E, elev. 872 m, 24 Apr. 2015, KYI-2015001 (holotype HHU; isotypes HHU, KB).

#### Perennial herbs.

Small, tender, hermaphroditic. Roots fibrous. Flowering stem erect, 2–6 cm long, pilose, light green or reddish to purple, with 2–5 sterile branches arising from base; sterile branches creeping after fruiting, elongated over 30 cm, 2 or more times branched at axils, densely pilose. Leaves opposite, basal and cauline, simple, estipulate, petiolate. Basal leaves 1 or 2 pairs, withered before flowering. Leaves on flowering stem, 1 pair, attached at 1/2 or below of the stem; petioles 1–5 mm, pilose; blade 2–5 × 2–8 mm, flabelliform, apex subtruncate to rounded, base attenuate, margins obscurely undulate to crenate or distinctly obtusely dentate (3–6 teeth), translucent white or brown ciliate, both surfaces glabrous. Leaves on sterile branches with long internode (to 8 cm at fruiting); petioles 2–12 mm, pilose; blade to 2 × 2.5 cm, suborbicular or widely ovate (upper ones), flabellate (lower ones), apex rounded, base cuneate, margins crenate with 5–10 flat obscure teeth on each side, translucent white or brown ciliate, upper surface glabrous, densely silvery dotted, pale green, lower surface pilose on veins, greenish-grey. Inflorescence 4- to 9-flowered cyme, surrounded by leaf-like bracts; pedicels ca. 1 mm, sparsely pilose. Bracteal leaves yellowish-green during flowering, turning to light green or green after fruiting; petioles 1–3 mm, pilose; blades 2–6 × 2–10 mm, obdeltoid, upper surface glabrous, densely silvery dotted, lower surface glabrous, greenish-grey, margins obscurely undulate to crenate or distinctly obtusely dentate, 2–5 teeth, translucent white or brown ciliate, obtuse to subtruncate at apex, base narrowly cuneate to cuneate. Flowers tetramerous; sepals 4, free, petaloid, 1 pair overlapping the other in bud, erect, yellow, widely ovate to widely subelliptic, ca. 2.5–3 × ca. 2 mm, glabrous, 3-veined, persistent, apex obtuse to truncate, slightly recurved; petals absent; stamens 8, in 2 section, ca. 1.3 mm, shorter than sepal; filaments filiform, 0.8–0.9 mm long; anthers yellow, 2-locular, 0.45–0.5 mm long, longitudinally dehiscent; pistil 2-carpellate, semi-inferior, ovary 1-locular, ovules at 2 parietal placentae, styles 2, free, ca. 1 mm long, stigma round, disc absent. Fruit capsule, pale green, glabrous, ca. 5.5 mm long, 2-lobed (horn shaped), lobes dehiscent along adaxial suture, slightly unequal; seeds numerous, dark brown, ellipsoid, with a carina on one side, thick-walled, 0.8–1.0 × 0.65–0.75 mm, with hemispheroidal tubercles, tubercles ca. 15 μm in diameter, seed surface covered with minute deciduous papillae.

#### Distribution.

*Chrysospleniumramosissimum* is only known to exist on Mt. Seonjaryeong in Gangwon-do, Korea, at an elevation of 630–910 m. To date, only one population of approximately 2,000 individuals has been discovered, near a small creek. In the absence of additional data, we presently score it as Data Deficient (DD), according to the IUCN Red List criteria (IUCN 2001).

#### Ecology.

*Chrysospleniumramosissimum* occurs in deciduous forests of mountain valleys, where it grows in humid and semi-shaded areas near small creeks along with *Quercusmongolica* Fisch. ex Ledeb., *Fraxinusrhynchophylla* Hance and *Acerbuergerianum* Miq. The flowering period of this species is late March to early May and the fruiting period is late May to early July.

#### Etymology.

The specific epithet of the new species refers to the highly branched sterile branches after fruiting.

#### Additional specimens examined (paratype).

SOUTH KOREA. Gangwon-do: Mt. Seonjaryeong, Hoenggye-ri, Daegwallyeong-myeon, Pyeongchang-gun, 37°41'25.80"N 128°45'27.22"E, elev. 872 m, 24 Apr. 2015, *KYI-2015002* (HHU), *KYI-2015003* (HHU), *KYI-2015004* (HHU), *KYI-2015005* (HHU), *KYI-2015006* (HHU); 37°41'33.65"N 128°45'25.26"E, elev. 872 m, 16 Apr. 2016, *KYI-2016001* (HHU), *KYI-2016002* (HHU), *KYI-2016003* (HHU), *KYI-2016004* (HHU), *KYI-2016005* (HHU), *KYI-2016006* (HHU).

##### Key to taxa of *Chrysosplenium* section *Pilosa* modified from Hara (1957)

**Table:** 

1	Sepals white. Anthers dark red	**2**
–	Sepals yellow or greenish. Anthers yellow	**3**
2	Stamens longer than or equal to sepals. Ovary superior. Seeds tuberculate	*** C. album ***
–	Stamens shorter than sepals. Ovary subsuperior. Seeds smooth	*** C. hebetatum ***
3	Sterile branches often hypogeous, filiform, with bulbil at top	*** C. maximowiczii ***
–	Sterile branches epigeous without bulbil	**4**
4	Seeds without tubercules	**5**
–	Seeds with tubercules	**6**
5	Leaves of sterile branches congested at distal end, with white variegated veins on upper surface	*** C. flaviflorum ***
–	Leaves of sterile branches distantly arranged, with silvery dotted upper surface	*** C. epigealum ***
6	Seed tubercles arranged without or on inconspicuous longitudinal ridges	**7**
–	Seed tubercles arranged on prominent longitudinal ridges	**9**
7	Leaves of sterile branches densely ciliate	*** C. villosum ***
–	Leaves of sterile branches rarely ciliate	**8**
8	Sterile branches highly (more than two times) branched, ca. 30 cm long after fruiting. Leaves of sterile branches with silvery dots, upper surface glabrous. Bracteal leaves yellowish-green	*** C. ramosissimum ***
–	Sterile branches unbranched, less than 15 cm long after fruiting. Leaves of sterile branches without silvery dots, upper surface pilose. Bracteal leaves bright yellow	*** C. valdepilosum ***
9	Basal leaves persistent after flowering	**10**
–	Basal leaves withered before flowering	**12**
10	Sepals yellow. Stamens shorter than sepals	*** C. sphaerospermum ***
–	Sepals light green. Stamens equal to or longer than sepals	**11**
11	Stamens equal to or slightly longer than sepals. Ovary 1/2 or 1/3 inferior	*** C. rhabdospermum ***
–	Stamens longer than sepals. Ovary 1/4 inferior or nearly superior	*** C. pseudopilosum ***
12	Leaves of sterile branches distantly arranged after fruiting. Bracteal leaves golden yellow, yellowish-green or green at flowering	**13**
–	Leaves of sterile branches congested at distal end after fruiting. Bracteal leaves green	**14**
13	Leaves of sterile branches pilose. Bracteal leaves golden yellow at flowering	*** C. aureobracteatum ***
–	Leaves of sterile branches glabrous. Bracteal leaves yellowish-green to green at flowering	*** C. pilosum ***
14	Seeds ca. 720 × 640 μm, with ca. 18 ridges, densely papillate	*** C. barbatum ***
–	Seeds ca. 640 × 510 μm, with ca. 16 ridges, sparsely papillate	*** C. fulvum ***

## Notes

It is noteworthy that *C.valdepilosum* and *C.ramosissimum* are sympatric in the type locality. The former species occupies moist soil at the side of a creek, while the latter inhabits damper parts closer to the main stream. The two species exhibit a high degree of morphological similarity upon flowering but can be distinguished by several characters, including the colour of the bracteal leaves at flowering, the vestiture of the leaves of sterile branches and the excrescence of the seeds (Table 1).

**Table 1.** Comparison of the key features of *C.ramosissimum* and *C.valdepilosum*.

Character	* C. ramosissimum *	* C. valdepilosum *
Sterile branches after fruiting	branched more than two times, ca. 30 cm long	unbranched or rarely branched, > 15 cm long
Size of sterile branch leaf blades after fruiting	up to 2 × 2.5 cm	up to 2.5 × 2.6 cm
Upper surface of sterile branch leaves	silvery dotted, glabrous	silvery dots absent, pilose
Bracteal leaves during flowering	Yellowish-green	bright yellow
Seed surfaces	tubercles ca. 15 μm in diam.	tubercles ca. 10 μm in diam.

It appears that *C.ramosissimum* and *C.valdepilosum* have not been recognised as different lineages until recently due to their sympatric distribution and high morphological affinity. Ignoring the importance of the sterile branch development pattern after fruiting may have been the main cause for the delay of the discovery of the new lineage. Further research on the genetic diversity and discovery of additional populations are necessary for the conservation of *C.ramosissimum*, an endemic species with a very narrow distribution.

*C.ramosissimum* may also be similar to *C.ramosum* due to its highly-branching habit. *C.ramosum* is also distributed in northeast Asia, including Korea, but belongs to the section *Oppositifolia* and differs in its spreading sepals (vs. erect) and smooth seeds (vs. tuberculate).

## Supplementary Material

XML Treatment for Chrysosplenium ramosissimum
